# Reliability and reproducibility of the new AO/OTA 2018 classification system for proximal humeral fractures: a comparison of three different classification systems

**DOI:** 10.1186/s10195-020-0543-1

**Published:** 2020-03-12

**Authors:** Giuseppe Marongiu, Lorenzo Leinardi, Stefano Congia, Luca Frigau, Francesco Mola, Antonio Capone

**Affiliations:** 1 Orthopaedic Clinic, Department of Surgical Sciences, Cagliari State University, Lungomare Poetto 12, 09126 Cagliari, Italy; 2 Department Economics and Business Science, Cagliari State University, Cagliari, Italy

**Keywords:** Proximal humeral fracture, Classification, Neer, AO/OTA, Reliability, Reproducibility, Interobserver agreement, Intraobserver agreement

## Abstract

**Background:**

The classification systems for proximal humeral fractures routinely used in clinical practice include the Neer and Arbeitsgemeinschaft für Osteosynthesefragen/Orthopaedic Trauma Association (AO/OTA) 2007 systems. Currently used systems have low inter- and intraobserver reliability. In 2018, AO/OTA introduced a new classification system with the aim of simplifying the coding process, in which the Neer four-part classification was integrated into the fracture description. The aim of the present work is to assess the inter- and intraobserver agreement of the new AO/OTA 2018 compared with the Neer and AO/OTA 2007 classifications.

**Materials and methods:**

A total of 116 radiographs of consecutive patients with proximal humeral fracture were selected and classified by three observers with different levels of experience. All three observers independently reviewed and classified the images according to the Neer, AO/OTA 2007, and new AO/OTA 2018 systems. To determine the intraobserver agreement, the observers reviewed the same set of radiographs after an interval of 8 weeks. The inter- and intraobserver agreement were determined through Cohen’s kappa coefficient analysis.

**Results:**

The new AO/OTA 2018 classification showed substantial mean inter- (*k* = 0.67) and intraobserver (*k* = 0.75) agreement. These results are similar to the reliability observed for the Neer classification (interobserver, *k* = 0.67; intraobserver, *k* = 0.85) but better than those found for the AO/OTA 2007 system, which showed only moderate inter- (*k* = 0.57) and intraobserver (*k* = 0.58) agreement. The two more experienced observers showed better overall agreement, but no statistically significant difference was found. No differences were found between surgical experience and agreement regarding specific fracture types or groups.

**Conclusions:**

The results showed that the Neer system still represents the more reliable and reproducible classification. However, the new AO/OTA 2018 classification improved the agreement among observers compared with the AO/OTA 2007 system, while still maintaining substantial descriptive power and simplifying the coding process. The universal modifiers and qualifications, despite their possible complexity, allowed a more comprehensive fracture definition without negatively affecting the reliability or reproducibility of the classification system.

Level of evidence: Level III, diagnostic studies

## Introduction

Proximal humeral fractures account for about 5.7% of all adult fractures [1] and, with a progressive increase of incidence with ageing, represent the most common fractures in patients older than 65 years [2, 3]. The majority of proximal humerus fractures are minimally displaced, but approximately 15–20% have more variable and complex patterns.

To improve the understanding and management of proximal humeral fractures, different classification systems are routinely used in clinical practice. The Neer classification system, updated in 2002 [4, 5], describes the effect of displacement forces exerted on the fracture fragments by their musculotendinous attachments, identifying 4 main fragments and 16 fracture subtypes. The AO/OTA classification system, based on the original Müller classification and updated in 2007 [6, 7], emphasizes the progressive severity of the fracture pattern with special attention to the integrity of the vascular supply, identifying three main fracture types which are then categorized into subgroups based on the degree of displacement, impaction, and dislocation of fracture fragment, resulting in a total of 27 fracture subtypes.

Although these two systems are the most commonly used, the reliability and reproducibility of the Neer and AO/OTA classification systems is still debatable. In literature, the interobserver reliability of the Neer classification system ranges widely from poor to substantial (kappa coefficient 0.21–0.77), while the intraobserver reliability is somewhat better (*k* = 0.5–0.8) [8–12]. On the one hand, the AO/OTA classification is considered more comprehensive [13], but on the other, the large number of subtypes could result in even poorer reliability and reproducibility, with interobserver agreement ranging from 0.11 to 0.65 [10, 11, 14]. However, these differences in terms of superiority among the two systems have not been fully clarified.

Due to the complexity of proximal humeral fracture patterns, the observed variability may be attributed to the difficult interpretation of tridimensional (3D) fractures on two-dimensional plain radiographs [15]. Poor-quality radiographs, osteoporotic bone, and overlapping fracture lines are factors hindering efforts towards a concrete classification. However, even the use of volumetric diagnostic tools, such as computerized tomography (CT) and 3D CT, did not substantially improve the reliability and reproducibility of the classification systems [16].

In 2018, the AO/OTA introduced a new fracture and dislocation classification compendium [17] with the aim of addressing the criticisms of the existing classification systems and simplifying the coding process. The new classification system integrates Neer’s criteria into the fracture description and consists of 13 fracture subgroups [18]. The compendium also introduces universal modifiers and qualifications into the classification descriptive terms, providing optional details about fracture morphology, displacement, and associated injury.

The aim of the present study is to assess the inter- and intraobserver reproducibility of the new AO/OTA 2018 classification compared with the Neer and AO/OTA 2007 classification systems. The secondary aim is to evaluate whether reliability and reproducibility differ with different levels of observer experience.

## Materials and methods

Radiographs of 136 consecutive patients treated for proximal humeral fracture in our department between January 2015 and December 2016 were selected from our hospital’s radiology picture archiving and communication system (PACS). Institutional review board approval and consent from patients participating in this study were obtained. One author (A.C.), who was not an observer in this study, screened all radiographs. Inclusion criteria were: male or female patients with proximal humeral fracture with at least an anterior–posterior projection in the scapular plane and an axillary view. Exclusion criteria were: patients with radiographs in only one view, patients without good-quality radiographs, and patients with previous proximal humeral fracture on the same side. Therefore, 116 out of the set of 136 radiographs were eventually selected for the review process. Scapular outlet views were also available in 21 patients. Nineteen patients had computed tomography (CT) scans, although these were not used for the evaluation. After anonymization, radiographs were arranged randomly for evaluation using a web-based list randomizer (https://www.random.org) and then imported into a Digital Imaging and Communications in Medicine (DICOM) medical imaging viewer (Horos v.3.3.5; The Horos Project). The viewer provides measurement adjustment tools such as zooming and panning, brightness and contrast windows, and angle measurements. Equivalent viewing conditions for the three observers were guaranteed by using the same workstation.

### Observers

Radiographs were evaluated by three observers with different levels of experience: an orthopedic resident who is receiving specific training in shoulder surgery, a junior shoulder surgeon, and a senior shoulder surgeon. Observers were familiar with the Neer and AO/OTA 2007 classification systems, using them in their daily clinical practice. The three observers and the nonobserver author jointly discussed the features of the AO/OTA 2018 system prior to the study.

### Study procedure

All three observers independently reviewed and classified 116 proximal humeral fractures according to the full Neer (17 categories), full AO/OTA 2007 (27 categories), and AO/OTA 2018 (13 categories) systems. An overview of the classification system, with pictures and description, was provided to all observers during the sessions [7, 17, 18]. Observers received a digital folder containing the anonymized DICOM files of each case, which they then imported into the DICOM viewer. Each observer reported results in a prefilled spreadsheet, which was then delivered to one of the authors (L.F.) responsible for the statistical analysis. Observers were not allowed to discuss their observations with the other investigators. To determine the intraobserver agreement, the observers performed a second review at least 8 weeks after the first session. At that time, the set of radiographs had been randomized to minimize any chance of recollection. All three observers completed the classification of the fractures in a mean time of 8.5 days (7–11 days; *p* > 0.05) for each session.

### Classifications

The Neer classification defines a four-segment system according to the number of displaced segments or parts, with additional categories for articular fractures and dislocations [4, 5]. The potential segments involved are greater tuberosity, lesser tuberosity, articular surface, and humeral shaft. A segment is defined as displaced when separation greater than 1 cm or angulation greater than 45° is present. The Neer classification system describes a total of 16 fracture categories.

The AO/OTA 2007 classification is based on the severity and articular/extraarticular and unifocal/bifocal pattern of the fracture, defining three main types (A, B, and C): type A fractures are extraarticular and unifocal, type B fractures are extraarticular and bifocal, and type C fractures are articular [6, 7]. Overall, the OTA/AO classification system for proximal humeral fractures has nine groups (11-A1/2/3, 11-B1/2/3, 11-C1/2/3). All groups are divided into three subgroups based on the degree of displacement, impaction, or dislocation, resulting in a total of 27 subgroups.

The AO/OTA 2018 classification maintains the original principles of the previous system with regard to definitions and the basic coding system [17]. Neer’s criteria were integrated into the fracture description for proximal humeral fractures to facilitate clinician comprehension of the terms unifocal and bifocal fractures [18]. This resulted in a simplified classification system, with three main types (A, B, and C): type A are extraarticular, unifocal, two-part fractures; type B are extraarticular, bifocal, three-part fractures; type C are articular or four-part fractures. A total of 13 potential subgroups are identified. The descriptive power of the AO system is guaranteed by the presence of the “universal modifiers” and “qualifications” that allow a useful characterization of the fracture pattern.

### Statistical analysis

Inter- and intraobserver agreement were determined through kappa value analysis [19]. The kappa coefficient (*k*) quantifies the absolute agreement of observers, accounting for the agreement that would occur by chance alone, as described by Cohen [20]. The *k* coefficient ranges from 1 (perfect agreement) to < 0 (no more agreement than would be expected by chance alone). The generated *k* values were interpreted according to the criteria of Landis and Koch [21]: ≥ 0.81, almost perfect agreement; between 0.61 and 0.80, substantial agreement; between 0.41 and 0.60, moderate agreement; between 0.21 and 0.40, fair agreement; and ≤ 0.2, slight agreement. Nonweighted *k* coefficients were used to determine interobserver reliability. Overall *k* ranges among the three observers were calculated using the mean *k* value for each of the three comparisons between two of three observers. The *k* values for intraobserver agreement were calculated for each of the individual observers before calculating the mean kappa value. The *k* values were classified according to Landis and Koch, as described above. Kappa coefficients were calculated for the full Neer classification, the full AO/OTA 2007 classification, and the full AO/OTA 2018 classification with and without the use of the universal modifiers and qualifications.

Mean *k* coefficients were compared using the standard Student *t*-test, with a significance level of *p* < 0.05 and 95% confidence interval (CI). All statistical analyses were performed using R software version 3.6.0.

## Results

The mean age of the patients was 64.3 years (45–78 years), and 74 out of 116 (63.8%) were female. According to the Neer classification, 78.7% of the fractures were one-part (15.5%) and two-part (63.2%) fractures. The most commonly identified pattern (mean value among the observers) was the two-part surgical neck fracture (51.1%). According to the AO/OTA 2007 system, 56.3% type A fractures and 33.9% type B fractures were found. The B1.1 type was the most frequent (26/116, 22.4%). Similarly, with the AO/OTA 2018 classification, type A fractures accounted for 58.6% (68/116), type B for 24.6%, and type C for 16.6%. The A2.1 (unifocal, two-part simple fracture of the surgical neck) represented 33%, and the B1.1 type (bifocal, three-part fracture of the surgical neck with greater tuberosity fracture) 23.85%, being the most frequent patterns. The most frequently used universal modifier was 5a, which corresponds to anterior dislocation and was found in 7 out of 116 fractures (6%). An overview of fracture types identified by the three observers according to the Neer, AO/OTA 2007, and new AO/OTA 2018 classifications for proximal humeral fractures is included in additional file 1.

### Interobserver reliability

An overview of the interobserver agreement of the Neer, AO/OTA 2007, and new AO/OTA 2018 classifications for proximal humeral fractures between the three observers is presented in Tables 1, 2, 3, and 4.

**Table 1:** Level of agreement among observers for the Neer classification after both review sessions

Neer	Observers	% agreement	Kappa (95% CI)	Judgement
First reading	1 versus 2	76.7	0.656 (0.550–0.763)	Substantial agreement
	1 versus 3	86.2	0.806 (0.722–0.891)	Almost perfect agreement
	2 versus 3	75.9	0.653 (0.547–0.759)	Substantial agreement
	Mean	79.6	0.705 (0.550–0.891)	Substantial agreement
Second reading	1 versus 2	69	0.556 (0.447–0.665)	Moderate agreement
	1 versus 3	81	0.742 (0.649–0.834)	Substantial agreement
	2 versus 3	72.4	0.607 (0.498–0.717)	Substantial agreement
	Mean	74.1	0.635 (0.447–0.834)	Substantial agreement

**Table 2:** Level of agreement among observers for the AO/OTA 2007 classification after both review sessions

AO/OTA 2007	Observers	% agreement	Kappa (95% CI)	Judgement
First reading	1 versus 2	65.9	0.680 (0.546–0.817)	Substantial agreement
	1 versus 3	60.3	0.561 (0.472–0.662)	Moderate agreement
	2 versus 3	58.6	0.546 (0.448–0.644)	Moderate agreement
	Mean	61.6	0.590 (0.448–0.817)	Moderate agreement
Second reading	1 versus 2	56	0.519 (0.420–0.617)	Moderate agreement
	1 versus 3	65.5	0.626 (0.534–0.718)	Substantial agreement
	2 versus 3	52.6	0.482 (0.383–0.582)	Moderate agreement
	Mean	58	0.542 (0.383–0.718)	Moderate agreement

**Table 3:** Level of agreement among observers for the AO/OTA 2018 classification after both review sessions

AO/OTA 2018	Observers	% agreement	Kappa (95% CI)	Judgement
First reading	1 versus 2	61.2	0.519 (0.413–0.625)	Moderate agreement
	1 versus 3	83.6	0.799 (0.717–0.881)	Substantial agreement
	2 versus 3	77.6	0.719 (0.625–0.813)	Substantial agreement
	Mean	74.1	0.679 (0.413–0.881)	Substantial agreement
Second reading	1 versus 2	62.1	0.534 (0.429–0.638)	Moderate agreement
	1 versus 3	81	0.769 (0.684–0.855)	Substantial agreement
	2 versus 3	75.9	0.7 (0.605–0.795)	Substantial agreement
	Mean	73	0.667 (0.429–0.855)	Substantial agreement

**Table 4:** Level of agreement among observers for the AO/OTA 2018 classification with the universal modifiers and qualifications after both review sessions

AO/OTA 2018 (with universal modifiers and qualifications)	Observers	% agreement	Kappa (95% CI)	Judgement
First reading	1 versus 2	53.4%	0.48 (0.382–0.578)	Moderate agreement
	1 versus 3	79.3%	0.767 (0.685–0.849)	Substantial agreement
	2 versus 3	70.7%	0.673 (0.581–0.764)	Substantial agreement
	Mean	67.8%	0.660 (0.382–0.849)	Substantial agreement
Second reading	1 versus 2	52.6%	0.472 (0.375–0.569)	Moderate agreement
	1 versus 3	77.6%	0.748 (0.664–0.832)	Substantial agreement
	2 versus 3	68.1%	0.644 (0.551–0.737)	Substantial agreement
	Mean	66.1%	0.621 (0.375–0.832)	Substantial agreement

After the first evaluation, the overall interobserver agreement was substantial for both the full AO/OTA 2018 classification (*k* = 0.68, 95% CI 0.41–0.81) and AO/OTA 2018 classification with the universal modifiers (*k* = 0.66, 95% CI 0.38–0.76). The interobserver agreement was substantial also for the full Neer classification (*k* = 0.70, 95% CI 0.55–0.89), while it was moderate for the full AO/OTA 2007 classification (*k* = 0.59, 95% CI 0.44–0.81). After the second evaluation, the overall interobserver agreement was lower for all the classifications. However, the differences between the kappa coefficient values of the first and second evaluations were statistically significant only for the Neer (*p* = 0.012) and AO/OTA 2007 (*p* = 0.020) classifications, while the differences for both versions of the AO/OTA 2018 classification (with and without the use universal modifiers) were not statistically significant. The mean overall interobserver agreement for the Neer and AO/OTA 2018 classifications was significantly higher than that for the AO/OTA 2007 classification (Table 5).

**Table 5:** Comparison of *k* coefficient values for interobserver agreement

Classification system	Mean *k* values	Difference in *k*	95% CI	*p*-Value
Neer versus AO/OTA 2007	0.670 versus 0.569	0.101	0.448–0.891	< 0.05
AO/OTA 2018 versus Neer	0.673 versus 0.670	0.003	0.413–0.891	> 0.05
AO/OTA 2018 versus AO/OTA 2007	0.673 versus 0.569	0.104	0.448–0.881	< 0.05
AO/OTA 2018 versus AO/OTA 2018 (with universal modifiers)	0.673 versus 0.640	0.033	0.382–0.881	> 0.05

Data acquired from interobserver testing from mean *k* values of two readings (Tables 1, 2, 3, and 4)

According to the specific experience of the three raters, better agreement between the two more expert evaluators was observed. Although the *k*-value was consistently approximately 0.1 points higher, no statistically significant difference was found. No differences were found between surgical experience and agreement regarding specific fracture types or groups.

### Intraobserver reliability

The three observers repeated the classification after a mean of 67 days (60–73 days). The overall intraobserver agreement was substantial (*k* = 0.75) for the AO/OTA 2018 system, both with and without the use of universal modifiers (Table 6). The Neer classification showed almost perfect intraobserver agreement among all three observers (*k* = 0.85, 95% CI 0.71–0.99). The reproducibility for the AO/OTA 2007 classification was only moderate (*k* = 0.58, 95% CI 0.50–0.69). The differences between the *k* values are presented in Table 7.

**Table 6:** Intraobserver reliability for individual reviewers for each system

Classification system	Observer	% agreement	Kappa (95% CI)	Judgement
Neer	Observer 1	87.1	0.801 (0.71–0.891)	Almost perfect agreement
	Observer 2	87.9	0.831 (0.75–0.912)	Almost perfect agreement
	Observer 3	95.7	0.941(0.891–0.991)	Almost perfect agreement
	Mean	90.2	0.857 (0.71–0.991)	Almost perfect agreement
AO/OTA 2007	Observer 1	63.8	0.597 (0.446–0.638)	Moderate agreement
	Observer 2	63.8	0.602 (0.508–0.696)	Substantial agreement
	Observer 3	57.8	0.542 (0.446–0.638)	Moderate agreement
	Mean	61.8	0.580 (0.446–0696)	Moderate agreement
AO/OTA 2018	Observer 1	81.9	0.770 (0.681–0.858)	Substantial agreement
	Observer 2	76.7	0.718 (0.627–0.809)	Substantial agreement
	Observer 3	81	0.767 (0.681–0.852)	Substantial agreement
	Mean	79.9	0.751 (0.627–0.858)	Substantial agreement
AO/OTA 2018 (with universal modifiers and qualifications)	Observer 1	79.3	0.770 (0.688–0.852)	Substantial agreement
	Observer 2	75.9	0.724 (0.641–0.815)	Substantial agreement
	Observer 3	80.2	0.777(0.696–0.858)	Substantial agreement
	Mean	78.5	0.752 (0.641–0.858)	Substantial agreement

**Table 7:** Comparison of *k* coefficient values for intraobserver agreement

Classification system	Difference in *k*	95% CI	*p*-Value
Neer versus AO/OTA 2007	0.277	0.446–0.991	< 0.05
AO/OTA 2018 versus Neer	0.103	0.627–0.991	> 0.05
AO/OTA 2018 versus AO/OTA 2007	0.171	0.446–0.858	< 0.05
AO/OTA 2018 versus AO/OTA 2018 (with universal modifiers)	–0.007	0.627–0.858	> 0.05

Data acquired from interobserver testing from mean *k* values of two readings (Tables 1, 2, 3, and 4)

The more experienced evaluators obtained better intraobserver agreement for all the classification systems. In particular, for the Neer classification, the intraobserver agreement was significantly better for the senior shoulder surgeon (observer 3) (*k* = 0.94 versus 0.81, *p* < 0.05) than for the other two observers.

## Discussion

An ideal fracture classification system should be reliable and reproducible and, moreover, a flexible evolving system which responds to user feedback originating from clinical practice and research. The Neer classification is the most commonly used system in current clinical practice, and although some authors have emphasized the usefulness of Neer’s criteria in intraoperative decision-making, it is generally reported also to have suboptimal intra- and interobserver reliability [8, 11]. The aim of the AO/OTA 2007 classification for humeral fractures was to provide a uniform and comprehensive coding system for fractures and dislocations, but due to its low reliability, reproducibility, and weak influence on the therapeutic choice, this system has not been completely validated. Therefore, ongoing concerns about terminology and the relevance of certain classification schemes resulted in the need to undertake the 2018 review [17]. In the AO/OTA 2018 classification system for proximal humeral fractures, the number of categories was reduced to 13 and Neer’s criteria were integrated into the fracture description to facilitate clinician comprehension of the terms unifocal and bifocal fractures. The intention of the AO/OTA review committee was to ensure consistency and provide greater clinical utility in fracture and dislocation classification. To the best of the authors’ knowledge, there are no studies in literature to date investigating the application of the AO/OTA 2018 classification for humeral proximal fractures. Therefore, the aim of the present study is to assess the reliability and reproducibility of the new AO/OTA 2018 classification compared with the two systems mainly used in clinical practice, viz. the Neer and AO/OTA 2007 classification systems.

In the present work, the new AO/OTA 2018 classification showed substantial mean inter- (*k* = 0.67) and intraobserver agreement (*k* = 0.75). These results were similar to the reliability observed for the full Neer classification (interobserver, *k* = 0.67; intraobserver, *k* = 0.85) but better than that observed for the full AO/OTA 2007, which showed only moderate inter- (*k* = 0.57) and intraobserver agreement (*k* = 0.58). The interobserver agreement of both the Neer and AO/OTA 2007 systems resulted slightly superior to the majority of those previously reported in literature, which in most cases ranged between fair and moderate [8–14, 19]. However, other researchers, such as Gumina et al. (*k* = 0.77) [11] and Sidor et al. (*k* = 0.80) [22], have reported substantial agreement between observers with specific experience in shoulder surgery.

A few studies have compared the reliability of the AO/OTA 2007 and Neer systems, with discordant reports in terms of the difference in inter- and intraobserver agreement between the two systems. In 1993, Siebenrock and Gerber [14] stated that the AO/OTA system (*k* = 0.53) had better reproducibility than the Neer system (*k* = 0.40), even if both achieved only moderate agreement. They concluded that neither the Neer nor AO/OTA 2007 classification was sufficiently reproducible to allow meaningful comparison of similarly classified fractures. According to a study by Sukthankar et al. in 2013 [23], the Neer system (*k* = 0.44) had slightly lower interobserver agreement than the AO/OTA system (*k* = 0.47). More recently, Papakostantinou et al. [8] reported slightly better results for interobserver agreement for the full Neer classification system. Similarly, Gumina et al. [11] reported better reproducibility for the Neer classification (*k* = 0.77) than for the AO/OTA 2007 classification (*k* = 0.64). Although substantial interobserver agreement was reported, the authors stated that the two systems presented weak coherence and might lead to different treatment approaches for the same fracture, depending on the classification used [24].

One of the main reasons indicated as the cause for low reproducibility and reliability is the number of categories in the classification systems [25]; therefore, several authors have used simplified versions of the Neer and AO/OTA classifications to improve both the intra- and interobserver agreement. However, only slight or even no improvement has been reported in literature [26]. According to Sidor et al. [22] and Papakostantinou et al. [8], the simplification of the Neer classification system from 16 categories to 6 or 4 more general categories based on fracture type did not significantly improve either interobserver reliability or intraobserver reproducibility. The simplified AO/OTA classification has been applied more rarely. Majed et al. simplified the AO classification to three categories and achieved an interobserver kappa value of 0.30 compared with 0.11 for the full 27-category system [10]. Siebenrock and Gerber also demonstrated an improvement in agreement with the three-category system (*k* = 0.53) compared with the nine-category AO system (*k* = 0.42) [14]. No substantial improvement was shown by Papakostantinou et al. when simplifying the full AO/OTA 2007 classification system to the nine- or three-category systems [8].

The introduction into current practice of the new AO/OTA 2018 classification system could fulfill the need for simplification, while preserving adequate descriptive power. The new AO/OTA 2018 classification presents a lower number of categories than both the AO/OTA 2007 (27 categories) and Neer classification (17 categories). Moreover, Neer’s criteria seemed to be successfully integrated into the AO/OTA 2018 classification with good coherence between Neer and AO/OTA 2018 subgroups; For example, the number of two-part surgical neck fractures in the Neer classification basically corresponded to the A2.1, A2.2, and A2.3 type fractures in the AO/OTA 2018 (51.1% versus 48.85%, *p* < 0.05). When we used the universal modifiers in addition to the AO/OTA 2018 classification, we still observed substantial inter- and intraobserver agreement, even though the number of possible categories increased. Reproducibility and reliability benefited from all of these factors combined, resulting in a system consistent with the Neer classification. Nevertheless, the higher intraobserver agreement for the Neer classification could be related to the better knowledge and familiarity of the three observers with this system in their daily clinical practice.

Another factor which several authors have claimed could positively influence the agreement in classification of proximal humeral fractures is, in fact, experience in the field. The two more expert of our observers obtained higher inter- and intraobserver reliability, but the differences between the observers were not always statistically significant. Similar results have been reported by other studies, suggesting that, the more experienced the observers or the shoulder specialists examining the radiographs, the greater the reliability of the system [22, 26]. Moreover, preliminary education discussions seemed to be effective for Shrader et al. [27], who discussed the reasons for disagreement between observers and then created a series of learning points to improve the accuracy of subsequent radiographic assessment. However, training the observers was not proven to significantly improve reproducibility, as reported by Mellema et al. [26]. In our study, we obtained higher interobserver agreement after the first review of the images and lower after the second review. In our opinion, the positive effect on the agreement due to the preliminary discussion among the observers may not have had the same strength over time. Therefore, future consideration should be given to pursuing methods for increasing surgeon receptiveness to training.

The low quality of routinely executed x-rays is one of the causes of lack of fracture interpretation and appropriate classification [5], therefore a good-quality anterior–posterior projection on the scapular plane and an axillary view are considered the minimum required images [27]. Several authors have tried to improve the reliability and reproducibility of the Neer and AO/OTA classifications, adding lateral scapular projections to trauma series, with poor results [28, 29]. CT scans have greater analysis power than plain x-rays, particularly in three- or four-part fractures and in the presence of osseous overlap. Unfortunately, the majority of the studies that explored the opportunity of using CT scans to improve agreement in classification reported no significant results [27, 30]. Other authors have reported that the use of 3D-CT scans does not improve the reliability of either the Neer or AO/OTA classification over traditional CT [31, 32]. Given that advanced imaging modalities have not been shown to improve interobserver agreement, we compared the three classification systems using only x-rays. Nevertheless, CT scans and 3D reconstruction play a crucial role for valuable comprehension of the fracture and to plan a surgical approach in more complex cases [33]. Torrens et al. found that addition of 3D imaging of proximal humeral fractures significantly increased the number of surgical decisions when compared with radiographs alone or together with CT [34]. Future developments in diagnostic imaging include alternative tools such as 3D models and augmented reality (holography) [35]. Recently, Cocco et al. reported better inter- and intraobserver agreement when 3D-printed models of the fractures were used to classify according to the Neer and the AO/OTA systems, compared with traditional CT scans and 3D reconstruction [36, 37].

Some limitations and strengths of this study should be addressed. The first limitation is the nonrandomized design of the study and the low number of observers. In the future, randomized controlled trials including more patients and observers could provide more robust evidence. Another possible limitation of the study is the slight majority of simple fractures in the cohort (60% of type A fractures, according to the AO system), which could have positively affected both the inter- and intraobserver agreement. Several investigators have reported a dramatic difference in interobserver agreement in simple versus complex fractures [27]. The use of the kappa value for the agreement among observers is a matter of controversy because its values depend strongly on the distribution of cases among the various classification categories within a sample; therefore our results should have been compared only with similar samples [19]. In addition, one potential confounding aspect of this study would be that all three observers had a specific shoulder surgery experience, with long-term familiarity with Neer’s criteria; for this reason, the Neer classification could have emerged as the more reproducible and reliable. The AO/OTA 2018 classification system, which includes part of the Neer criteria, could have benefited from the expertise of the observers. However, the difference between the mean *k* values of the new AO/OTA 2018 and the AO/OTA 2007 classifications was statistically significant and showed a clear hierarchy in terms of reliability and reproducibility. The main strength is that, to date, no studies in literature have evaluated the inter- and intraobserver reproducibility for the new AO/OTA 2018 classification.

In conclusion, our results for the Neer system confirm the high values of reproducibility previously reported in literature. The new AO/OTA 2018 classification improves the agreement of observers compared with the AO/OTA 2007 system, while still maintaining substantial descriptive power, and seems to address part of the criticism of the codifying process. The universal modifiers and qualifications, despite their possible complexity, allow a more comprehensive fracture definition without negatively affecting the reliability and reproducibility of the classification process. Issues about the definition of displacement remains, as the inability to differentiate the pattern of minimally displaced fractures, often arbitrary for the observer, is still a concern. In the future, new classification systems should be able to assess proximal humeral fracture patterns using 3D models and relating them to bony landmarks and soft tissue attachments, with the aim of providing precise quantification of fragments and displacement, and clear indications for treatment. A randomized controlled trial with a larger number of observers recruited following a power study would provide more robust results for the reliability and reproducibility of this common injury and widely used classification systems.

## Supplementary information

**Additional file 1: Supplementary data.** Spreadsheets showing fractures types distribution among the 3 observers at the first and second observation.

## Data Availability

The datasets used and analyzed during the current study are available from the corresponding author on reasonable request.

## Abbreviations

AO/OTA: Arbeitsgemeinschaft für Osteosynthesefragen/Orthopaedic Trauma Association
CT: computed tomography
DICOM: Digital Imaging and Communications in Medicine

## References

[1] Court-Brown CM, Caesar B (2006). Epidemiology of adult fractures: a review. Injury.

[2] Tarantino U, Capone A, Planta M (2010). The incidence of hip, forearm, humeral, ankle, and vertebral fragility fractures in Italy: results from a 3-year multicenter study. Arthritis Res Ther.

[3] Passaretti D, Candela V, Sessa P, Gumina S (2017). Epidemiology of proximal humeral fractures: a detailed survey of 711 patients in a metropolitan area. J Shoulder Elb Surg.

[4] Neer CS (1970). Displaced proximal humeral fractures. I. Classification and evaluation. J Bone Joint Surg Am.

[5] Neer CS (2002). Four-segment classification of proximal humeral fractures: purpose and reliable use. J Shoulder Elb Surg.

[6] Müller ME, Koch P, Nazarian S, Schatzker J (1990). The comprehensive classification of fractures of long bones.

[7] Marsh JL, Slongo TF, Agel J (2007). Fracture and dislocation classification compendium—2007: orthopaedic trauma association classification, database and outcomes committee. J Orthop Trauma.

[8] Papakonstantinou MK, Hart MJ, Farrugia R (2016). Interobserver agreement of Neer and AO classifications for proximal humeral fractures. ANZ J Surg.

[9] Cuny C, Baumann C, Mayer J (2013). AST classification of proximal humeral fractures: introduction and interobserver reliability assessment. Eur J Orthop Surg Traumatol.

[10] Majed A, Macleod I, Bull AMJ (2011). Proximal humeral fracture classification systems revisited. J Shoulder Elb Surg.

[11] Gumina S, Giannicola G, Albino P (2011). Comparison between two classifications of humeral head fractures: Neer and AO-ASIF. Acta Orthop Belg.

[12] Iordens GIT, Mahabier KC, Buisman FE (2016). The reliability and reproducibility of the Hertel classification for comminuted proximal humeral fractures compared with the Neer classification. J Orthop Sci.

[13] Court-Brown CM, Garg A, McQueen MM (2001). The epidemiology of proximal humeral fractures. Acta Orthop Scand.

[14] Siebenrock KA, Gerber C (1993). The reproducibility of classification of fractures of the proximal end of the humerus. J Bone Jt Surg.

[15] Carofino BC, Leopold SS (2013). Classifications in brief: the Neer classification for proximal humerus fractures. Clin Orthop Relat Res.

[16] Sjödén GOJ, Movin T, Aspelin P (1999). 3D-radiographic analysis does not improve the Neer and AO classifications of proximal humeral fractures. Acta Orthop Scand.

[17] Meinberg E, Agel J, Roberts C (2018). Fracture and dislocation classification compendium—2018. J Orthop Trauma.

[18] Humerus J Orthop Trauma 32:S11–S20. 10.1097/BOT.0000000000001062.

[19] Audigé L, Bhandari M, Kellam J (2004). How reliable are reliability studies of fracture classifications? A systematic review of their methodologies. Acta Orthop Scand.

[20] Cohen J (1960). A coefficient of agreement for nominal scales. Educ Psychol Meas.

[21] Landis JR, Koch GG (1977). The measurement of observer agreement for categorical data. Biometrics.

[22] Sidor ML, Zuckerman JD, Lyon T (1993). The Neer classification system for proximal humeral fractures: an assessment of interobserver reliability and intraobserver reproducibility. J Bone Joint Surg Am.

[23] Sukthankar AV, Leonello DT, Hertel RW (2013). A comprehensive classification of proximal humeral fractures: HGLS system. J Shoulder Elb Surg.

[24] Congia S, Palmas A, Marongiu G, Capone A (2019). Is antegrade nailing a proper option in 2- and 3-part proximal humeral fractures?. Musculoskelet Surg.

[25] Brorson S, Olsen BS, Frich LH (2012). Surgeons agree more on treatment recommendations than on classification of proximal humeral fractures. BMC Musculoskelet Disord.

[26] Mellema JJ, Kuntz MT, Guitton TG, Ring D (2017). The effect of two factors on interobserver reliability for proximal humeral fractures. J Am Acad Orthop Surg.

[27] Shrader MW, Sanchez-Sotelo J, Sperling JW (2005). Understanding proximal humerus fractures: image analysis, classification, and treatment. J Shoulder Elb Surg.

[28] Sidor ML, Zuckerman JD, Lyon T (1994). Classification of proximal humerus fractures: the contribution of the scapular lateral and axillary radiographs. J Shoulder Elb Surg.

[29] Sallay PI, Pedowitz RA, Mallon WJ (1997). Reliability and reproducibility of radiographic interpretation of proximal humeral fracture pathoanatomy. J Shoulder Elb Surg.

[30] Bernstein J, Adler LM, Blank JE (1996). Evaluation of the Neer system of classification of proximal humeral fractures with computerized tomographic scans and plain radiographs. J Bone Jt Surg.

[31] Bruinsma WE, Guitton TG, Warner JJP, Ring D (2013). Interobserver reliability of classification and characterization of proximal humeral fractures. J Bone Jt Surg Ser A.

[32] Berkes MB, Dines JS, Little MTM (2014). The impact of three-dimensional CT imaging on intraobserver and interobserver reliability of proximal humeral fracture classifications and treatment recommendations. J Bone Jt Surg - Am.

[33] Marongiu G, Mastio M, Capone A (2013). Current options to surgical treatment in osteoporotic fractures. Aging Clin Exp Res.

[34] Torrens C, Marí R, Cuenca M (2018). 3D reconstruction does not improve agreement and results in an increase in surgical indications in proximal humeral fractures. J Orthop.

[35] Lal H, Patralekh MK (2018). 3D printing and its applications in orthopaedic trauma: a technological marvel. J Clin Orthop Trauma.

[36] Cocco LF, Yazzigi JA, Kawakami EFKI (2019). Inter-observer reliability of alternative diagnostic methods for proximal humerus fractures: a comparison between attending surgeons and orthopedic residents in training. Patient Saf Surg.

[37] Marongiu G, Congia S, Verona M (2018). The impact of magnetic resonance imaging in the diagnostic and classification process of osteoporotic vertebral fractures. Injury.

